# Role of Procalcitonin and C-reactive Protein as Predictors of Sepsis and in Managing Sepsis in Postoperative Patients: A Systematic Review

**DOI:** 10.7759/cureus.31067

**Published:** 2022-11-03

**Authors:** Junaid Hassan, Safeera Khan, Rukhe Zahra, Abdul Razaq, Ali Zain, Laiba Razaq, Mahrukh Razaq

**Affiliations:** 1 General Surgery, M. Islam Medical & Dental College/M. Islam Teaching Hospital, Gujranwala, PAK; 2 Internal Medicine, California Institute of Behavioral Neurosciences & Psychology, Fairfield, USA; 3 Family Medicine, Quaid-e-Azam Medical College, Bahawalpur, PAK; 4 Urology, District Headquarters Hospital Teaching Hospital, Faisalabad, PAK; 5 Specialized Health Care and Medical Education, Gujranwala Medical College, Gujranwala, PAK; 6 Internal Medicine, Akhtar Saeed Medical and Dental College, Lahore, PAK; 7 Obstetrics and Gynecology, Tehsil Headquarter Hospital, Gujranwala, PAK

**Keywords:** c-reactive protein (crp), postoperative infection, procalcitonin (pct), pct, systemic inflammatory response syndrome (sirs), sirs, sepsis

## Abstract

Systemic inflammatory response syndrome (SIRS) and sepsis are inflammatory responses to infection or trauma, causing symptoms and adverse outcomes such as organ shutdown and death. Different scoring systems can help in the diagnosis of SIRS and sepsis. Several biomarkers such as C-reactive protein (CRP), procalcitonin (PCT), and white blood cells (WBCs) can serve as predictors of sepsis. Surgery, trauma, and burns are the non-inflammatory causes of SIRS and sepsis. In postoperative patients, both inflammatory and non-inflammatory causes of immune response may co-exist. The role of inflammatory biomarkers in identifying sepsis development, deciding to use antibiotics, and discharging patients needs further exploration and clarity. We searched medical databases such as PubMed/Medline, PMC, ScienceDirect, Cochrane Library, and Google Scholar for relevant medical literature. The identified papers were screened, eligibility criteria were applied, and 15 research papers were identified. The finalized papers explored the roles of CRP and PCT in postoperative patients. Both CRP and PCT are raised in a postoperative patient, and then, gradually, the levels decrease. However, in case of an infection, these levels continue to rise and signify an infection, which may progress to sepsis. The cut-off values can guide decision-making about when to start antibiotics and discharge the patient. PCT was found to be more reliable in identifying the infection and preventing the development of sepsis. Further research is needed to identify the exact cut-off values that can help in decision-making.

## Introduction and background

Sepsis is one of the leading causes of death worldwide. According to a global burden of disease study, there were 48.9 million cases and 11 million deaths worldwide in 2017 [1]. In the United States, sepsis is one of the major causes of in-hospital deaths and costs more than 24 billion USD annually [2]. Out of all in-hospital deaths, postoperative sepsis is one of the main causes of organ dysfunction [3]. Sepsis, a life-threatening condition leading to septic shock and organ dysfunction, occurs due to the host’s excessive or disturbed immune response to the infection. It is one the most common causes of death worldwide in postoperative patients and is considered a significant healthcare burden. Systemic inflammatory response syndrome (SIRS) is an inflammatory response to several infectious and non-infectious causes such as trauma, burns, and surgery. Infection is characterized by either a fever of more than 38°C or hypothermia of less than 36°C, along with tachycardia, tachypnea, and leukocytosis. If there is a presence of infection along with these symptoms, it is referred to as sepsis.

Surgery, trauma, and burns are non-infectious causes and can lead to SIRS; however, patients with these conditions or those undergoing surgery are also prone to developing infections and sepsis. Patients who undergo emergency surgery are more prone to sepsis than those undergoing elective surgery. Several indicators or biomarkers have been used to predict the prognosis and outcome of surgery in postoperative patients. Acute Physiology and Chronic Health Evaluation (APACHE) II, Simplified Acute Physiology Score (SAPS3), and Sequential Organ Failure Assessment (SOFA) with specific criteria are widely used to predict outcomes in postoperative patients [4]. Several other biomarkers such as interleukin-1 (IL-1), white blood cell (WBC) count, C-reactive protein (CRP), procalcitonin (PCT), and blood lactate levels have also been used. These predictors correlate directly with the outcomes of patients and have been considered independent predictive factors compared to APACHE, SOFA, and SAPS3 scoring [4]. However, CRP, PCT, and WBC count are commonly used to detect postoperative complications and guide patient management. They can also be raised because of non-inflammatory causes such as surgery, considering any postoperative complication may induce SIRS. However, an infection increases this risk several times. The risk of postoperative complications and sepsis is around 5-10% higher in emergency surgery than in elective surgery [5]. Patients undergoing emergency surgery are eight times more likely to develop complications and sepsis and have fatal outcomes [6].

Biomarkers such as PCT and CRP can not only be used in predicting sepsis in postoperative patients but can also predict the outcome of septic patients. Early recognition can help improve the overall prognosis of patients by assisting the diagnostic and management approach in surgical intensive care unit (ICU) patients. Treatment in surgical ICUs should not just be based on clinical knowledge about procedures, techniques, and protocols. It should also focus on safety, cost-effectiveness, and the outcomes of critically ill patients.

CRP, an acute phase reactant, acts as a biomarker that is raised if a person develops an infection or is septic. It can get elevated within hours of the trigger and reach maximum levels within 48 hours [7]. It is considered a significant marker in acute infections and can be used to identify early-onset sepsis in postoperative patients [8]. PCT, a protein, is another biomarker that can be detected in blood if there is a systemic inflammatory reaction. PCT can be helpful in the diagnosis and assessment of the severity of sepsis. Patients suffering from burns or trauma or those who have undergone surgery can have raised CRP in the acute phase and increased CRP levels in the blood, which in healthy individuals may not be high. Because surgery is considered trauma, and if it leads to sepsis, it will be a non-inflammatory cause; however, if it is complicated by sepsis, it can lead to septic shock. PCT and CRP can predict sepsis; however, their role in postoperative patients is less explored.

In this systematic review, we aim to explore the diagnostic role of PCT in postoperative septic patients and patients with trauma. We also aimed to explore its role in the prognosis of sepsis and management and how the levels of CRP and PCT can help with the duration of antibiotic use and the overall prognosis of the patient.

## Review

Methodology

This systemic review was conducted using the Preferred Reporting Items for Systemic Review and Meta-Analysis (PRISMA) 2020 guidelines [9].

Search Sources and Strategy

We searched PubMed, PubMed Central (PMC), Medline, Cochrane Library, ResearchGate, and ScienceDirect to search for the relevant literature. We used various combinations of CRP, PCT, sepsis, and postoperative period to search all databases. In PubMed, however, along with these keywords, the following strategy was developed and used to search relevant literature in PubMed’s MeSH database: ((((“Procalcitonin/chemistry”[Mesh]) OR (“C-Reactive Protein/blood”[Mesh])) AND (“Systemic Inflammatory Response Syndrome”[Mesh])) OR (“Sepsis”[Mesh])) AND (“Postoperative Period”[Mesh]). Table 1 shows the databases used and the identified numbers of papers for each database.

**Table 1:** Keywords/Strategy used and the number of identified papers.

Keywords/Search strategy	Database used	Number of results
((((“Procalcitonin/chemistry”[Mesh]) OR (“C-Reactive Protein/blood”[Mesh])) AND (“Systemic Inflammatory Response Syndrome”[Mesh])) OR (“Sepsis”[Mesh])) AND (“Postoperative Period”[Mesh])	PubMed MeSH database	185
CRP OR Procalcitonin AND Sepsis OR SIRS AND Postoperative period	PubMed	252
CRP OR Procalcitonin AND Sepsis OR SIRS AND Postoperative period	PMC/Medline	415
CRP AND Procalcitonin AND Sepsis AND Postoperative period	ScienceDirect	470
Procalcitonin AND Postoperative period	Cochrane Library	15
Total number of research papers identified		1,337
Number of articles after removing duplicates		689

Inclusion and Exclusion Criteria

We selected the latest literature and the articles published in the past five years, including papers written in the English language, or if the full-text English-language translation is available. We only included research papers involving human participants.

Articles were excluded if the full text of the papers could not be retrieved. Articles focussing on CRP and PCT in non-surgical causes of sepsis were also not included. Gray literature and proposal papers were also not included.

Selection Process

We transferred the shortlisted articles to Endnote and removed any duplicate papers. Each article was screened through titles and abstracts and independently assessed by JH and SK (first and second authors). In case of a conflict about eligibility, the concerns were discussed with all other co-authors and finalized by mutual consensus. The shortlisted articles were further evaluated by evaluating the full text, and only relevant articles were assessed. Inclusion and exclusion criteria were applied, and only articles that satisfied the criteria were shortlisted.

Quality Assessment of the Studies

The shortlisted articles were checked for quality using the relevant quality appraisal tools. All co-authors were involved in quality checks. Observational studies were assessed for quality using the Newcastle-Ottawa tool, while systematic reviews were evaluated using the Assessment of Multiple Systematic Review (AMSTAR) tool. The Scale for the Assessment of Narrative Review (SANRA) was utilized for narrative reviews. Only studies that satisfied the quality appraisal were included in the systematic review.

Data Collection Process

After the articles were finalized for the systematic review and extracted, the primary outcomes were assessed along with other necessary information. JH and SK independently extracted the data, and all authors were equally involved in finalizing the retrieved data and outcomes observed using the data extraction questionnaires.

Results

Study Identification and Selection

**Figure 1:**
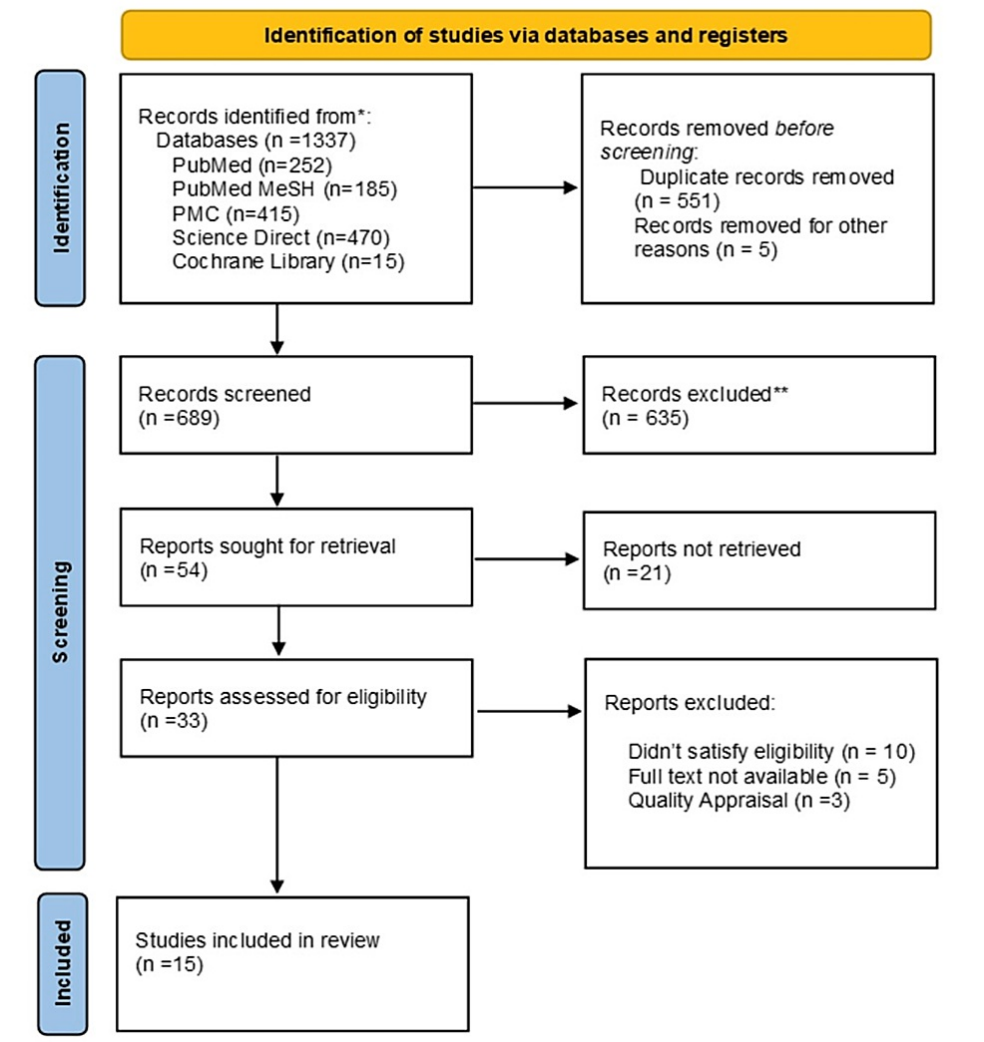
PRISMA flowchart showing the process of article selection. PRISMA: Preferred Reporting Items for Systemic Review and Meta-Analysis

We identified a total of 1,337 relevant articles using all databases. In total, 551 duplicate articles were removed before screening them in detail. After screening these articles by going through titles and abstracts and retrieving full texts, 33 articles were shortlisted. The shortlisted full-text articles were assessed for eligibility and quality, and 15 articles were finalized for review. The selection process of the studies is shown in Figure 1 in the PRISMA flowchart.

The articles were assessed for eligibility using the relevant quality appraisal tools. Table 2 shows the results of the quality appraisal.

**Table 2:** Quality appraisal using the Newcastle-Ottawa tool.

Study	Selection	Comparability	Outcome
Arbutina et al. 2022 [10]	****	*	***
Mahmoud et al. 2022 [11]	****	*	***
Franeková et al. 2021 [12]	****	*	**
Xu et al. 2018 [13]	****	**	*
Li et al. 2018 [14]	****	*	**
Khambalia et al 2018 [15]	****	**	***
El Zaher et al. 2022 [16]	****	*	**
Sariego-Jamardo et al. 2017 [17]	****	**	*
Amanai et al. 2022 [18]	****	*	***
Spoto et al. 2018 [19]	****	**	***
Chomba et al. [20]	****	*	**

Outcomes Measured

The primary outcomes extracted from the finalized research papers were the role of CRP and PCT levels, development of SIRS, sepsis, or death. The secondary outcomes assessed were stay in the surgical ICU and the overall outcome of monitoring CRP and PCT levels. A few studies explored the effect of immunosuppressants on biomarkers and the development of SIRS or sepsis. The relative changes in biomarkers with immunosuppressant use were also observed.

Study Characteristics

We reviewed 15 research papers with a total of 3,484 postoperative patients. Out of these finalized studies, two were systematic reviews and meta-analyses [21,22], two were narrative reviews [23,24], one was a non-randomized clinical trial, and the remaining nine were observational studies [10-19], and one non-randomized interventional study [20]. All studies involved operated patients and the measurement of CRP and PCT postoperatively during their in-hospital/ICU stay and their role in patients developing sepsis and SIRS. One of the studies focused on the effect of immunosuppressants on the levels of PCT in postoperative patients. A total of 3,484 postoperative patients were reviewed in this systematic review. Deaths or other outcomes such as the number of ICU days and cases developing sepsis were not reported in all studies. Few studies compared the levels of CRP and PCT for different surgical procedures and whether it was clean or contaminated surgery. Studies that compared the group monitored with PCT or CRP levels with the non-monitored group showed relatively fewer days of ICU stay. Table 3 shows a summary and characteristics of all included studies.

**Table 3:** Summary of the included studies. SIRS: systemic inflammatory response syndrome; CRP: C-reactive protein; PCT: procalcitonin; WBC: white blood cell; IL-6: interleukin 6; SPKT: simultaneous pancreatic and kidney transplant; AL: anastomotic leak; PCNL: percutaneous nephrolithotomy

Authors and year of publication	Type of the study	Purpose of the study and biomarker studied	Number of participants	Results	Conclusions
Arbutina et al. 2022 [10]	Prospective cohort study	To study the role of PCT, CRP, and serum amylase in differentiating SIRS from sepsis	100	PCT and CRP can be utilized in distinguishing between SIRS and sepsis and are very valuable in the early diagnosis and differentiation of SIRS and sepsis in the postoperative period. However, in the rest of the postoperative period, they have less diagnostic accuracy	Identifying and utilizing the specific biomarker in the postoperative phase for the diagnosis of abdominal sepsis can be very helpful in differentiating between SIRS and sepsis
Mahmoud et al. 2022 [11]	Retrospective observational study	To determine the association between increased PCT and infectious complications in immunosuppressed postoperative liver transplant patients	60	Sixty postoperative patients were divided into positive and negative culture groups and followed up with TLC and PCT levels. TLC was elevated till the fourth postoperative day. PCT was also significantly higher in the positive culture group than in the negative one on the first, third, and fifth postoperative days	PCT level and TLC on the first postoperative day can detect sepsis and guide early antibiotic treatment after liver transplant. It will help pick up early infections and exclude infections in almost 83.7% of patients, thus avoiding unnecessary usage of empiric antibiotics
Franeková et al. 2021 [12]	Observational Study	To observe the biomarkers presepsin, PCT, CRP, WBC, and IL-6 in patients on immunosuppressants	140	Concentrations of presepsin and PCT were higher in patients receiving immunosuppressants than in the non-treatment group. In contrast, CRP was lower in the Tx group. Decreased CRP and IL-6 were found for all immunosuppressants, whereas PCT increased after anti-thymocyte globulin and corticosteroids	Different biomarkers responded differently in postoperative patients without any infectious complications. Adding immunosuppressants in post-transplant patients decreased CRP significantly compared to the postoperative patients not receiving immunosuppressants. PCT, however, was increased only after corticosteroids and anti-thymocyte globulin
Xu et al. 2018 [13]	Prospective observational study	To assess the predictive role of CRP/albumin ratio in PCNL patients	556	123 patients out of the 556 patients who underwent PCNL developed SIRS. Based on the analysis, female gender, positive urine culture, hs-CRP/albumin ratio, and neutrophil to lymphocyte ratio were independent predictors of post-PCNL SIRS. The optimal cut-off value of the hs-CRP/albumin ratio was 0.06	The preoperative hs-CRP/albumin ratio predicts sepsis better than other systemic inflammatory biomarkers. It is independently a predictor of the development of SIRS after PCNL
Li et al. 2018 [14]	Retrospective observational study	To determine the factors predicting the development of SIRS and sepsis in postoperative patients	337	59 patients developed postoperative fever, 22 developed SIRS, and two patients developed septic shock. Patients with SIRS showed high CRP and were associated with long surgical times. Predictive nomogram models showed the c-statistics of 0.766 and 0.887, respectively. With treatment, patients fully recovered	Factors like a high stone burden, long surgical time, positive stone culture, and diabetes mellitus increase the risk of fever or SIRS after RIRS for kidney stones. Increased CRP levels after the surgery also increases the chance of SIRS and sepsis. The developed nomogram can help predict sepsis and its outcomes
Khambalia et al 2018 [15]	Observational Study	To evaluate the changes in CRP and other biomarkers in omentum and serum in postoperative SPKT patients	46	Increased CRP levels were observed in the first three days after surgery and were significantly related to the Post-Operative Morbidity Survey.	The study suggested the predictive value of CRP in perioperative morbidity following SPKT within 72 hours
El Zaher et al. 2022 [16]	Prospective observational study	To evaluate the role of PCT, CRP, and WBC in predicting AL in colorectal surgery	205	This study included 205 patients; 22 patients had AL. Three days postoperatively, PCT was a better predictor for AL than CRP and WBC. The cut-off value for PCT of 4.93 ng/mL had the highest sensitivity, specificity, and negative predictive value on the fifth postoperative day. If PCT is combined with CRP and WBCs, it improves its predictability	CRP, PCT, and WBC levels over five postoperative days had a better predictive value than the single marker’s daily measurements. A combination of all three parameters can serve as a guide to discharge patients from the hospital early
Sariego-Jamardo et al. 2017 [17]	Prospective observational study	To evaluate the kinetics of PCT, CRP, and IL-6 in various surgeries in children	119	PCT, IL-6, and the CRP maximal peak occur quickly after surgery. The greatest elevation of biomarkers was seen after abdominal surgery and dirty surgery	PCT can serve as a useful tool for diagnosing hospital-acquired sepsis after surgery
Amanai et al. 2022 [18]	Prospective observational study	To evaluate the role of pre-pepsin in the early detection of infections and compare it to the role of CRP and PCT	114	114 patients were examined, of whom 27 developed infectious complications. CRP and PCT markedly increased from the first postoperative day to the third postoperative day, which then gradually decreased toward the sixth postoperative day. On the other hand, pre-pepsin did not show major changes after surgery, but it increased on the fourth and sixth postoperative days when the complications occurred	Serial measurements of pre-pepsin, CRP, and PCT trends after colorectal surgeries can help detect postoperative infectious complications
Jerome et al 2022 [21]	Systematic review and meta-analysis	To assess the diagnostic accuracy of PCT in liver transplant patients	363	Eight studies with 363 participants explored the diagnostic accuracy of PCT. PCT showed a sensitivity of 70% and a specificity of 77%	PCT is a moderately well diagnostic test in diagnosing postoperative infection/sepsis in liver transplant patients
Jerome et al. 2022 [22]	Systematic review and meta-analysis	To assess the role of PCT and IL-6 in diagnosing sepsis in major hepatobiliary surgery	1,611 for diagnostic accuracy of PCT	12 articles were reviewed. Ten studies with 1,611 patients reported were assessed for the role of PCT. The sensitivity of PCT was higher in colorectal surgeries compared to upper gastrointestinal surgeries	Heterogenicity of the sampling and inconsistencies in the cut-off values are hindrances
Spoto et al. 2018 [19]	Observational study	To assess the role of PCT in post-surgical patients.	90	PCT values differed between post-surgical patients developing infections and those with no infections. PCT with a cut-off value of >1.0 ng/mL on the first two post-surgical days and >0.5 ng/mL on the third day resulted in the diagnosis of infection, whereas with a value <0.5 ng/mL on the fifth day, patients can be discharged	Daily PCT measurement in the postoperative period after abdominal surgery can represent a useful diagnostic tool for improving the outcomes
Chomba et al. 2020 [20]	Clinical trial	To analyze the use of the PCT algorithm in using antibiotic use in postoperative patients	80	Patients in the intervention group had more antibiotic-free days alive of 7.7 days compared to the control group’s mean of 3.8 days. The intervention group had a lower mortality rate than the control group	The duration of antibiotic treatment was not different between the two groups. However, the PCT group had more live antibiotic-free days and lower in-hospital mortality than the control group
Silosi et al. 2018 [23]	Narrative review	To evaluate the role of biomarkers like PCT in postoperative clinical settings	0	The differentiation of sepsis between infectious causes and non-infectious SIRS is difficult. There is a need to identify other accurate sepsis biomarkers that can be utilized along with the existing ones, such as CRP and PCT	There is no identified ideal biomarker that can aid in diagnosing sepsis. The discovery of new serum markers and their combinations with the existing biomarkers may prove to be better predictors for the diagnosis and prognosis of sepsis
Parli et al. 2018 [24]	Narrative review	To evaluate the role of PCT in trauma and emergency surgeries	0	PCT may help identify and diagnose infection in trauma and postoperative patients. However, because of the heterogeneity of patients, surgery and trauma may elevate PCT levels even without an infection	Although PCT levels and concentrations may provide insights into diagnosing infection, no standard approach can be recommended

Discussion

The postoperative period is a critical time where patient monitoring is crucial to ensure adequate patient recovery. If complicated with surgical-site infection, SIRS, or full-blown sepsis, the postoperative recovery is delayed or may lead to other adverse outcomes, including death. Several factors play a role in the poor postoperative outcome or prognosis, for example, if it is an emergency or elective surgery, comorbidities, or immunocompromised patients. These conditions make patients more prone to infections and sepsis. If the patient is adequately monitored and early infection can be identified, these untoward effects can be avoided. Septic biomarkers such as CRP and PCT are raised in response to surgical trauma and infection. If utilized, they may help diagnose patients with SIRS and sepsis and may serve as a guide in managing them. However, the cut-off value and exploring the differences between their levels in surgical and non-surgical causes are important.

Pathophysiology of C-reactive Protein and Procalcitonin Concerning Sepsis

CRP is produced by the liver and is raised in response to cytokines triggered by inflammation. It is an acute phase reactant raised in the early phases of the infection. In response to bacterial infection, it rises after 12 hours, peaks after 36-50 hours, and has a moderate specificity for bacterial infections [25]. It is commonly used as a biomarker in the early stages of infection to screen infants for sepsis because of high sensitivity in the first 24 hours of life [8]. Although it has low specificity in adult sepsis, considering its early rise in levels in response to the trigger, it may be helpful in the first three postoperative days after surgery. This low specificity for bacterial infection, longer induction time, and suppression in response to corticosteroid use may limit its use as a reliable marker in postoperative patients on immunosuppression or those using corticosteroids [26].

**Figure 2:**
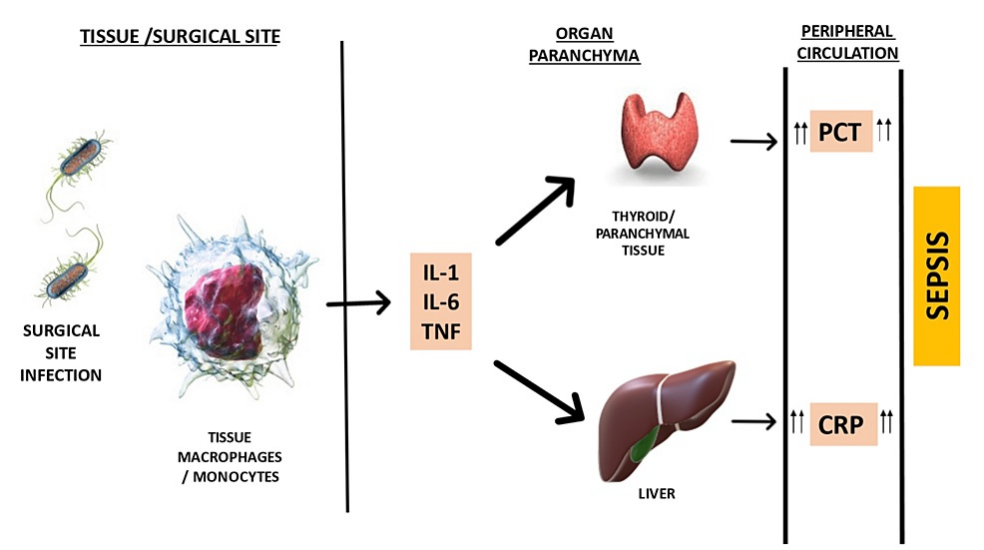
Mechanism of CRP and PCT production. IL-1: interleukin 1, IL-6: interleukin 6; TNF: tumor necrotic factor; PCT: procalcitonin; CRP: C-reactive protein Figure created by the corresponding author (SK).

PCT, a protein consisting of 116 amino acids used to identify and monitor sepsis, can be considered in postoperative settings. PCT is a precursor protein for calcitonin produced by the thyroid; calcitonin is a distinct protein like PCT. PCT rapidly processes to mature hormone calcitonin; therefore, the levels are very low at less than 0.1 µg/L. However, in the case of bacterial infection, PCT is induced outside the thyroid in other organs and parenchymal tissues and is released into circulation in large amounts. Figure 2 shows a brief overview of CRP and PCT production.

Role of the Type of Surgery on the Levels of Biomarkers in Predicting Sepsis

The risk factors vary according to the type of surgery. Elective surgeries generally have better outcomes because of proper preoperative preparation and prophylactic antibiotics. In contrast, in emergency surgeries, where comorbidities may not be well controlled, or there may not be adequate time to prepare the patient or for the prophylactic antibiotic to work, there are more chances of developing infections. An observational study involving 115 operated patients evaluated the changes in biomarkers in different types of surgeries [17]. SIRS in postoperative patients causes an elevation of CRP, PCT, and IL-6, which may differ according to the surgery type. This study showed that the biomarkers were higher in the case of abdominal surgery [17].

Another observational study by Franeková et al. involved 140 postoperative patients who underwent abdominal surgery, transplant, or major cardiac surgery. They divided the groups into two subsets, one undergoing major abdominal or cardiac surgeries and the other undergoing transplant [12]. The patients undergoing major abdominal and cardiac surgeries in the first subset showed elevated levels of biomarkers. The transplant patients were the treatment group and were given immunosuppressants. No infections were observed in the first week, all patients survived the first month, and none developed sepsis [12]. In post-transplant patients receiving immunosuppressants, CRP levels were lower. PCT, however, was still higher in response to surgical stress [12].

Similarly, the surgery site was also linked to different levels of biomarkers and accuracy in predicting infections. A systematic review by Jerome et al. to assess the role of PCT in abdominal surgeries showed that PCT was more effective in colorectal surgery than in upper gastrointestinal (GI) surgeries [22].

Role of C-reactive Protein and Procalcitonin in the Diagnosis of Infection and Sepsis in Postoperative Patients

In contrast to CRP, PCT has a rapid induction time of three to six hours, and high sensitivity and specificity for bacterial infection can make it a valuable biomarker in postoperative patients [26]. Its levels decrease in response to antibiotic treatment, which makes PCT an important and useful biomarker in managing postoperative ICU patients. It is synthesized in response to bacterial infections and not viral infections. The bacterial insult triggers cytokine-induced monocytes, which induce low PCT production within two hours [27]. These low levels play a role in the further synthesis of PCT in all storage tissues and cause a PCT surge with a peak in 12-24 hours, causing the levels to be as high as 100,000-fold than normal concentrations, with the surge continuing till the trigger exists [27].

Surgery or trauma can raise PCT levels independently of infection. Within 24 hours of surgery, there is an early rise in PCT, followed by a rapid decrease, which does not increase again in routine. However, in case of no infection, the elevated PCT levels in response to trauma/surgery return to baseline rapidly. In such cases, a second peak or rise in PCT levels can make an infection more likely. This makes it superior to CRP in identifying bacterial infection and sepsis in surgical patients, particularly postoperative patients. Sariego-Jamardo et al. showed that if PCT is raised after the initial 24 hours of the surgery, it has a significant clinical correlation; however, the role of CRP after 24 hours is not as clear [17]. PCT is less affected by trauma associated with surgery or other non-inflammatory causes.

Arbutina et al., in an observational study conducted in Serbia, showed the role of PCT and CRP in differentiating sepsis from SIRS [10]. The study involved 100 postoperative patients who underwent abdominal surgeries because of acute abdomen secondary to secondary peritonitis. They chose patients with more than 12 hours of pathological perforations of the gallbladder, stomach, intestines, appendix, or complications arising after surgeries. Abdominal sepsis was defined as a culture-positive infection of the peritoneal cavity. Patients were divided into the SIRS group and the sepsis group [10]. They collected blood samples before surgery and every day after surgery. CRP and PCT were significantly higher in the sepsis group than in the SIRS group. However, none of the biomarkers had any discriminant value postoperatively [10].

Khambalia et al. observed 46 postoperative patients who underwent simultaneous pancreas and kidney transplantation (SPKT) to see the evolution of biomarkers including IL-6, IL-10, and CRP in SIRS [23]. They observed 46 out of 69 recipients who developed SIRS blood and omental samples. CRP levels were raised and statistically related to the adverse outcomes of the SPKT surgery [15]. CRP levels serve as an acute phase reactant and can be a predictor in the early postoperative days. However, The CRP/albumin ratio instead of CRP levels alone can be used as a risk factor or a predictor of mortality at 90 days in septic patients as it can be used as a long-term prognostic biomarker [23]. In a retrospective observational study, Xu et al. used CRP/albumin ratio in SIRS patients to assess its predictive value [13]. They included 556 postoperative patients who underwent percutaneous nephrolithotomy. Of these, 123 developed SIRS, and CRP/albumin ratio was found to have statistically significant and better predictive value than other observed biomarkers and factors [13].

Role of Procalcitonin in Managing Sepsis in Postoperative Patients

**Figure 3:**
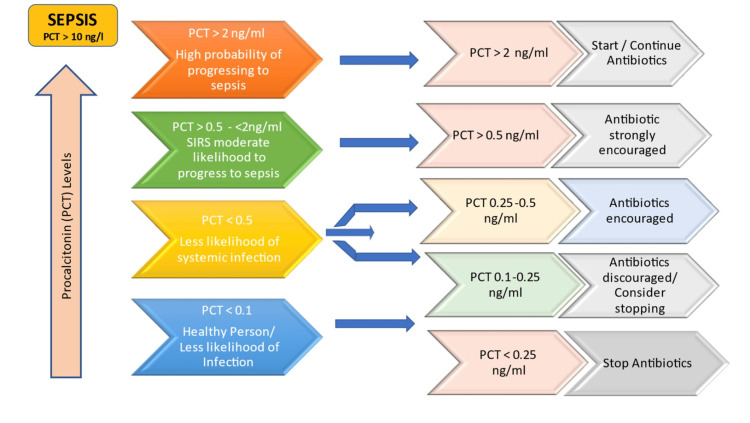
PCT as a guide in patient management. PCT: procalcitonin Figure created and owned by the corresponding author (SK) based on the information gathered.

PCT levels of more than 2 µg/L indicate an increased probability of SIRS and systemic bacterial infection, whereas levels of <0.5 µg/L make infection less likely, and, hence, the patient can be discharged. Figure 3 shows the levels of PCT and its role in managing patients.

Table 4 shows the number of participants and the identified variables from the finalized studies.

**Table 4:** Summary of variables identified in selected studies. SIRS: systemic inflammatory response syndrome; PCT: procalcitonin; CRP: C-reactive protein

Study	Total number of patients undergoing surgery	Patients developing surgical-site infection/ SIRS	Biomarker assessed/Cut-off values	Patients developing sepsis	Average length of ICU stay	Outcomes/Deaths
Spoto et al. [19]	90	32 + 10		17		5
Chomba et al. [20]	80, 40 (PCT) 40 (Control)		PCT	14, 15	16–17 days	6, 12
Arbutina et al. [10]	100, 45 (SIRS) 55 (sepsis)	45	-	55	-	-
Mahmoud et al. [11]	60	17	PCT cut-off value >9 ng/mL	-	6–7 days	-
Li et al. [14]	337	59 (fever) + 22		2		
Franeková et al. [12]	Total 140, control group 86, treatment group 54	0–7 days	PCT >2.8 µg/L on the first postoperative day in more than half of treatment patients and only 10% of non-treatment patients	CRP is less in the treatment group than in the non-treatment group	7 days	-
Xu et al. [13]	556	123	CRP/Albumin ratio cut-off value = 0.06	22	17 days	-
Khambalia et al. [15]	46		CRP raised with postoperative morbidity	-	6.5 days	-
EL Zaher et al. [16]	205	14	PCT>CRP as diagnostic criteria for postoperative anastomotic leak	22		6
Sariego-Jamardo et al. [17]	123	-	PCT and CRP in dirty surgery > clean surgery cases	-	-	-
Amanai et al. [18]	114	27	CRP and PCT markedly increase on postoperative days 1–3 and then gradually decrease	-	-	-

A retrospective observational study involving 60 postoperative liver transplant patients admitted to the ICU was conducted to determine the association between increased levels of PCT and infection and related complications in immunosuppressed patients [11]. They also explored non-infectious causes of the transplant. After their cultures were taken in the initial postoperative days, they were divided into positive and negative culture groups. They found that PCT was significantly higher in the positive culture group than in the negative one on the first, third, and fifth postoperative days. CRP, on the other hand, although high, was not significantly raised in both groups. Considering CRP as an acute phase reactant, it reached its peak on the third postoperative day and then was lowered by the fifth day. PCT is a good marker to detect inflammation early; however, in postoperative patients, it may be affected by factors such as immunosuppression, surgical procedures, underlying conditions, and comorbidities [11]. Therefore, PCT in the first few days of surgery can serve as a guide to initiate early antibiotic therapy in high-risk patients.

Biomarkers such as CRP and PCT can not only serve as a guide to diagnose infection but also as a predictor to safely discharge patients. The changes in their levels over the initial postoperative days help identify potential cases of sepsis or the ones that can be discharged easily. El Zaheer et al. investigated the role of PCT, CRP, and WBC count in predicting the anastomotic leak (AL) after colorectal surgery [16]. They observed 205 patients who had colorectal surgery, of whom 22 had an AL between the sixth and 14th postoperative days. In total, 14 patients developed clinical signs of SIRS [16]. Six patients died because of sepsis and complications. The preoperative levels of CRP, PCT, and WBC were compared to the postoperative levels. A rise in CRP levels of 50 mg/L or more between two days was considered significant and had the greatest predictive value between the fourth and fifth postoperative days. In contrast, a rise in PCT levels of >0.5 ng/mL between any two consecutive days was considered a significant predictor of adverse outcomes. They found the levels to be significantly different between patients with ALs and those without them. Therefore, these biomarkers are a useful indicator of surgical complications such as AL and can be a good predictor for discharge [16].

Similarly, an observational study by Amanai et al. involved 114 participants, of whom 27 developed infectious complications [18]. The complications included intra-abdominal infections or urinary tract infections. They also found significant differences in CRP and PCT levels between infected and non-infected patients. The levels increased between the first postoperative day to the third postoperative day and then decreased toward the end of the sixth postoperative day. Their values were independently associated with the presence of infection and non-infectious patients [18]. Non-infectious patients were discharged on the sixth postoperative day. Patients who developed infections were diagnosed on the fifth postoperative day. The CRP levels were high on postoperative days two, three, and four, which can help diagnose infections early and prevent adverse outcomes [18]. Both these observational studies involved abdominal surgeries and had a good sample size, and both used serial measurements of the biomarkers to assess the outcomes. The greatest predictive value was found to be on the fourth or fifth postoperative day in both studies. A rising PCT value or, if not declining, 10% of the value per day is considered a poor prognostic sign. Another observational study by Spoto et al. included 90 patients subjected to major abdominal surgery to observe the role of PCT over three postoperative days. The levels of PCT were significantly different between infectious and non-infectious patients. A PCT level of >1.0  ng/mL on the first or second day after surgery was diagnostic of infections, whereas it was >0.5  ng/mL for the third day. On the fifth day, a value <0.5  ng/mL was used for the early discharge of patients in case of no infection [19].

Most studies showed significant differences between the levels of PCT and CRP between infectious and non-infectious patients, which may help identify infections. The cut-off values can guide identifying cases potentially developing serious complications so that antibiotics can be initiated early. However, Prali et al., in their narrative review, argued that despite their potential role in early identification and providing insights into infections, there is no standard consistent approach to their implications and applications [24]. Similarly, Silosi et al. concluded in their review that PCT and CRP were the main biomarkers that are mainly put to clinical use. However, they cannot accurately predict results because of their insignificant efficacy and sensitivity [23]. The systematic review by Jerome et al. to assess the role of PCT in liver transplant patients showed moderately well diagnostic accuracy. When evaluated in only liver transplant patients, the heterogenicity was reduced and showed that PCT had 70% sensitivity and 77% specificity [21].

Limitations

The identified studies were mainly observational and showed heterogenicity in terms of sample size, the cut-off values of the biomarkers, the timing of measurement of these biomarkers, and the use of immunosuppression in the case of transplants. Not all studies assessed the same variables and secondary outcomes.

## Conclusions

This systematic review was conducted to explore the role of CRP and PCT in the early detection of sepsis and in decision-making to start antibiotics or discharge patients. Based on the articles reviewed, these biomarkers can be used in predicting sepsis in postoperative patients and can also help in anticipating the outcomes of septic patients. Early recognition can help improve the overall prognosis of patients by assisting the diagnostic and management approach in surgical ICU patients. The rising levels of PCT beyond the cut-off value on the second, third, fourth, and fifth postoperative days can be a better indicator than CRP. Using these biomarkers will lead to fewer sepsis and related complications. However, because the cut-off values were not the same for different types of surgeries and other medications of the patients, including immunosuppression, the role of these biomarkers may vary in different conditions, and there is a need for proper guidelines and consistent cut-off values separately for patients on immunosuppressants or those not on immunosuppressants. More large-scale studies are needed to assess their role in specific types of surgeries to rule out heterogeneity and better predict their role in assessing sepsis.
